
JOURNAL INFORMATION
==============================
NLM Title Abbreviation: Behav Neurol
Journal ID: Behav Neurol
NLM Journal ID: 8914585
Journal ID: BN

Behavioural Neurology

ISSN: 0953-4180
EISSN: 1875-8584
Publisher: Wiley

ARTICLE INFORMATION
==============================
PMCID: PMC5434404
PMID: 21422543
DOI: 10.3233/BEN-2010-0324
Article ID: 461010
Article version: 1
Subjects: Introduction

Extended Abstracts from the Second Meeting of the Federation of European Societies of Neuropsychology

Vingerhoets Guy
Print publication date: 2010
Electronic publication date: 2011 Mar 7
Volume: 23
Issue: 4
First page: 161
Last page: 161
Received 2011 Jan 31; Accepted 2011 Jan 31
Copyright: Copyright © 2010 Hindawi Publishing Corporation and the authors.
Copyright year: 2010
License: This is an open access article distributed under the Creative Commons Attribution License, which permits unrestricted use, distribution, and reproduction in any medium, provided the original work is properly cited.
License URL: https://creativecommons.org/licenses/by/3.0/

==============================
Behavioural Neurology 23 (2010) 161
DOI 10.3233/BEN-2010-0324
IOS Press

Introduction

### Extended Abstracts from the Second Meeting of the Federation of European Societies of Neuropsychology

Guy Vingerhoets
*Chair of the Local Organising Committee*
*E-mail: guy.vingerhoets@ugent.be*

Dear colleague,

From 22–24 September 2010 the Second Meeting of the Federation of European Societies of Neuropsychology was held at the Royal Tropical Institute in Amsterdam, The Netherlands. The aim of the conference was to provide a meeting place for neuropsychologists, clinicians as well as researchers, from all over Europe. About 450 participants from 17 European countries gathered to present their research and exchange ideas on brain behaviour relationships. Some of the best contributions are presented here as extended abstracts in the proceedings of this meeting. We are grateful to the editors of Behavioural Neurology, Stefano Cappa and Argye Hillis-Trupe, for making this possible.

The Federation of the European Societies of Neuropsychology was founded in 2008 with the objective to further scientific and professional issues within the field of neuropsychology including cognitive neuropsychology, clinical neuropsychology, behavioural neurology, neuroimaging, and neuropsychological rehabilitation. The organisation of periodical scientific meetings is an important instrument to achieve that aim. The second meeting of our young Society was locally organised by the Dutch Neuropsychological Society, supported by the ESN-board , the ESN-scientific advisory board, and the Local Organising Committee. Many thanks to all of you who contributed to its success. I also would like to thank all participants who made a scientific contribution to this conference, either by poster, individual, or symposium presentation. I hope that you will enjoy browsing through these thematically organised proceedings and I certainly hope to see you again on our future meetings.

Yours sincerely,

ISSN 0953-4180/10/$27.50 © 2010 – IOS Press and the authors. All rights reserved
